# In Utero Exposure to Glucocorticoids and Pubertal Timing in Sons and Daughters

**DOI:** 10.1038/s41598-019-56917-7

**Published:** 2019-12-30

**Authors:** Sofie Aagaard Sand, Andreas Ernst, Lea Lykke Harrits Lunddorf, Nis Brix, Anne Gaml-Sørensen, Cecilia Høst Ramlau-Hansen

**Affiliations:** 1 Department of Public Health, Research Unit for Epidemiology, Aarhus University, Aarhus, Denmark; 2 Department of Urology, Aarhus University Hospital, Aarhus, Denmark

**Keywords:** Asthma, Epidemiology, Paediatric research, Reproductive signs and symptoms

## Abstract

Early pubertal timing has been associated with adult diseases, and identifying preventable causes is of importance. In utero exposure to exogenous glucocorticoids, has been associated with changes in the reproductive hormonal axes in the children, which may influence pubertal timing. Exogenous glucocorticoids can be indicated for diseases such as asthma, allergy, skin diseases, as well as muscle and joint diseases. The aim was to explore the association between in utero exposure to glucocorticoids and pubertal timing in the children. This population-based study was conducted in the Puberty Cohort including 15,819 children, which is a sub-cohort of the Danish National Birth Cohort. Information on maternal glucocorticoid treatment was collected through interviews during pregnancy. Information on pubertal timing was obtained by questionnaires every 6 months throughout puberty, including Tanner Stages, axillary hair, acne, voice break, first ejaculation and menarche. The potential impact of confounding by indication was explored by stratifying on indication and treatment status. Overall, 6.8% of the children were exposed to glucocorticoids in utero. Exposure to glucocorticoids in utero was not associated with earlier puberty for neither boys nor girls with combined estimates of 0.4 months (95% CI: –1.5; 2.2) and –0.7 months (95% CI: –2.5; 1.2).

## Introduction

A trend towards earlier pubertal timing has been observed in the Western world since the 19^th^ century. The trend is most prominent for girls, while it is more uncertain for boys due to the lack of data and the fact that male pubertal markers are difficult to accurately assess^1^. Genetics have a great impact on determining pubertal timing^2^. However, it cannot solely explain the apparent decline in pubertal timing as the genetic pool is considered to remain relatively stable. Obesity during childhood seems to explain part of the decline^3^. Endocrine disrupters^4^ and nutrition^5^ may as well contribute to this potential trend. Early puberty has been associated with diseases later in life including obesity, diabetes mellitus, cardiovascular diseases, breast cancer and testicular cancer^6^. Additionally, it has been associated with behavioural and emotional problems^7^. It is, therefore, of public health interest to identify potential causes of the decline in pubertal timing.

In utero exposures, such as medication, can impact the child’s health throughout life^8^. Medications containing glucocorticoids are commonly used for diseases, which also affect women in the fertile age, e.g. asthma, allergy, skin diseases, as well as more severe autoimmune diseases^9^. Studies have found an association between in utero exposure to glucocorticoids and altered hypothalamic-pituitary-adrenal-axis (HPA-axis) activity in infants and children^10^. Most studies have found decreased HPA-axis activity, which would be expected to accelerate pubertal timing through disinhibition of the hypothalamic-pituitary-gonadal-axis (HPG-axis)^11^. The HPG-axis, and indirectly the HPA-axis, as the two axes interact, regulate the initiation of pubertal timing, which makes a potential association between in utero exposure to glucocorticoids and pubertal timing plausible^12,13^.

The fetus is normally protected from large amounts of glucocorticoids by the placental enzyme, 11β-hydroxysteroid dehydrogenase type 2, however, the barrier may not be completely resistant^14^. In utero exposure to glucocorticoids has also been associated with metabolic- and endocrine diseases in the child^15^, which may be an intermediate factor on the path towards altered pubertal timing.

The aim of this study was to investigate the association between in utero exposure to exogenous glucocorticoids and pubertal timing in boys and girls in a large longitudinal cohort with multiple measurements of various markers of pubertal timing, while considering the indication for use of glucocorticoids in pregnancy.

## Materials and Methods

### Study population

**Figure 1:**
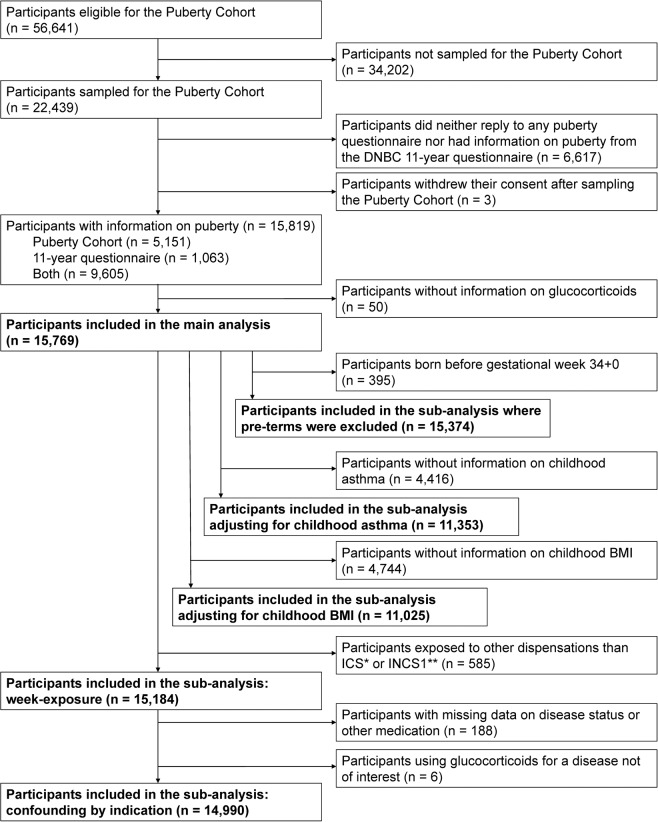
Flow chart of the participants in the Puberty Cohort. The number of participants in the different analyses is highlighted. *ICS: inhaled corticosteroids. **INCS1: first-generation intranasal corticosteroids.

The study was conducted in the Puberty Cohort, which is a sub-cohort in the Danish National Birth Cohort (DNBC)^16^. The Puberty Cohort consists of live-born singleton children born between 2000 and 2003 of mothers recruited to the DNBC (Fig. 1). In the DNBC, interviews on maternal lifestyle, health and socioeconomic factors during pregnancy were carried out at approximately gestational weeks 12 and 30. Follow-up, including questions on health of the children, continued after birth at age 6 and 18 months, as well as 7 and 11 years. Of 56,641 eligible children, 22,439 children were invited to participate in the Puberty Cohort. The participants were sampled from 12 subgroups based on pre- and perinatal exposures, thought to be of relevance for pubertal development to ensure a broad exposure contrast in the Puberty Cohort. A randomly selected group of 8,000 of the 56,641 eligible children was added. Additionally, among the sampled children, we considered those with information on pubertal timing from the 11-year questionnaire in the DNBC as participants in this study. In total, 15,819 children (70%) returned at least one puberty questionnaire, and 15,769 (99.7%) of those had information on maternal glucocorticoid treatment during pregnancy.

### Glucocorticoids

Information on glucocorticoid treatment was obtained through the two telephone interviews conducted during pregnancy. The women were asked about medication for specific diseases, including asthma, allergy, skin diseases and muscle or joint diseases (yes/no). If they replied *yes*, they were asked to report the pharmaceutical formulation and the specific drug name (either a list of often used medication was provided or the option to provide the name as free text). Further, they were asked about timing of their medication use in gestational weeks. Finally, the women were asked if they had taken any medication not yet mentioned. The questionnaires are available on DNBC’s website^17^.

The mothers were divided into three groups according to the reported formulation of glucocorticoids in order to make the most homogenous treatment groups: (1) Inhaled corticosteroids (ICS) and first-generation intranasal corticosteroids (INCS). (2) Second-generation INCS, cream, tablets, injections, suppositories, eye- and eardrops. (3) Non-treated (reference group). First generation INCS have a systemic absorption similar to ICS^18,19^ and were therefore grouped together, whereas second-generation INCS have a lower systemic absorption, and were, therefore, in a separate group^20^. A priori, it was expected that ICS and INCS were used every day as treatment guidelines recommend this^21^, which further makes the first treatment group more homogenous. The women were registered as treated independently of the duration of the treatment period, and more than one type of glucocorticoid used in the same week, did not double the exposure in the analyses.

To explore the risk of confounding by indication, information on diseases, for which glucocorticoids may be indicated, was obtained, including asthma, allergy, skin diseases and muscle or joint diseases. The women were divided into four groups based on their disease- and medication status: (1) Women with no disease and no treatment with glucocorticoids were included as the reference group. (2) Women with a disease who received no treatment. (3) Women with a disease who received treatment that did not contain glucocorticoids. (4) Women with a disease who received treatment containing glucocorticoids.

### Puberty

Information on pubertal timing was obtained through half-yearly online questionnaires from 11.5 years of age until the child reached full sexual maturity or 18 years of age, whichever came first. The questions were based on a questionnaire used in the Avon Longitudinal Study of Parents and Children^22^. Additionally, information on pubertal timing was obtained from the 11-year questionnaire in the DNBC. The children were asked to evaluate their current stage of puberty on several pubertal markers, including genital hair and genital- and breast development according to Tanner Stages using short text descriptions and illustrations^23,24^, axillary hair (yes/no), acne (yes/no), voice break (yes – definitive (adult voice), yes – sometimes (voice break), no), first ejaculation (yes/no, age), and menarche (yes/no, age). When the child reached one pubertal marker, this specific question was not included in the next questionnaire. The questionnaire is available on DNBC’s website^17^.

### Covariates

Directed acyclic graphs (DAGs)^25^ (Supplementary Fig. 1) were used to identify potential confounders in the study, which included maternal age, maternal body mass index (BMI), maternal age at menarche, smoking during first trimester and highest social class of the parents. Information on maternal age was obtained from the Danish Medical Birth Register, information on highest social class of the parents was obtained from Statistics Denmark and the remaining covariates were available from the DNBC questionnaires. For sub-analyses, length of gestation was obtained from the Danish Medical Birth Register, and childhood asthma was obtained from the 7-year questionnaire within the DNBC.

### Statistical analyses

Data on pubertal markers were left, right or interval censored as information on current pubertal stage was reported half-yearly. Some had already reached different pubertal markers before returning the first questionnaire, which make these data left censored. Data on pubertal markers reached between two questionnaires were interval censored, and data on pubertal markers that were not obtained at the last questionnaire were right censored. Therefore, the data was analysed using a censored regression model from which the mean age difference (in months) at attaining the different pubertal markers was estimated for each exposure group with the unexposed as the reference group. The regression model assumed a normal distribution of age at attaining the given pubertal markers. This statistical strategy is recommended in studies when analysing pubertal development with multiple longitudinally collected measurements^26^. Hence, residuals were checked for normality in R (x64 3.3.1) by comparing the non-parametric cumulative incidence function based on the Turnbull Estimator, against the normal distribution. Moreover, a combined estimate for pubertal timing was estimated using a model for the combined association with all pubertal markers for each sex by using Huber-White robust variance estimation to handle multiple testing of correlated outcomes^27,28^. All analyses were stratified by sex. The statistical package, -intreg- in Stata/MP 15.1 (StataCorp LLC, College Station, Texas), was used to analyse data.

Sampling weights were applied to account for the non-random sampling of participants. Selection weights were used to overcome the risk of selection bias due to non-participation, where the prior mentioned covariates were included, as well as the exposure variable. Sampling and selection weights were inverse probability weights that were multiplied and used to reweight the analyses. Robust standard errors were included due to the use of sampling and selection weights, as well as clustering of siblings^29,30^.

Different sub-analyses were performed. First, to examine possible dose-response effects, a sub-analysis was performed, where the number of gestational weeks treated with ICS or first-generation INCS was included as a continuous variable in units of five weeks up to gestational week 28. In total, 15,184 participants were included in this analysis. Second, to explore the risk of confounding by indication, women who were treated with glucocorticoids for a disease not of interest (n = 6), women who were treated with other formulations than ICS or first-generation INCS (n = 585), and women with missing data in either disease status or medication use (n = 188) were not included in the analysis. In total, 14,990 participants were included in this analysis. Third, to consider the risk that glucocorticoids given to women at risk of preterm birth, children born before week 34 + 0 (n = 395) were excluded from the main analysis. At last, additionally adjustments were performed to explore childhood asthma by adjusting for a dichotomous childhood asthma variable (yes/no) and also to explore childhood BMI, the children’s BMI obtained from the 7-year questionnaire were included.

### Ethical approval

All procedures performed in this study involving human participants were in accordance with the ethical standards of the institutional and/or national research committee (The Committee for Biomedical Research Ethics in Denmark has approved data collection in the DNBC ((KF) 01-471/94). A written informed consent was obtained from mothers at recruitment including both mother’s and child’s participation until the children turned 18 years of age. This study was approved by the steering committee of the DNBC (2012-04, 2015-47 and 2017-06) and the Danish Data Protection Agency (2012-41-0379 and 2015-57-0002). As data to the study was gathered and approved prior to this study, further approval was not needed.

## Results

In total, 15,769 (70%) mother-child pairs were included in the main analysis. Of these, 6.8% were exposed to glucocorticoids in utero, with the most common being ICS (27.8%), first-generation INCS (19.7%) and cream (43.6%). The reported frequencies of different diseases of interest among mothers were asthma 6.4%, allergy 14.6%, skin diseases 6.8% and muscle or joint diseases 11.5%. As expected, mothers reporting use of glucocorticoids were more likely to have a disease of interest compared to non-treated (Table 1). A large number of women reported one of the diseases, but did not report any treatment for it (17.3%).

**Table 1:** Maternal characteristics according to glucocorticoid treatment during pregnancy in the Puberty Cohort.

Glucocorticoids in pregnancy	None	ICS^1^/INCS1^2^	Other^3^	Total
n (%)	14691 (93.2)	493 (3.1)	585 (3.7)	15769 (100.0)
Pre-pregnancy BMI^4^, mean (sd)	23.8 (4.5)	24.5 (5.1)	24.2 (4.9)	23.8 (4.6)
Missings, n (%)	195 (1.3)	10 (2.0)	10 (1.7)	215 (1.4)
Maternal age at delivery in years, mean (sd)	30.6 (4.4)	31.1 (4.6)	31.0 (4.3)	30.6 (4.4)
Missings, n (%)	6 (0.04)	0 (0.0)	0 (0.0)	6 (0.04)
Maternal age of menarche, n (%)				
Earlier than peers, n (%)	3681 (25.1)	142 (28.8)	166 (28.4)	3989 (25.3)
Same time as peers, n (%)	8379 (57.0)	260 (52.7)	330 (56.4)	8969 (56.9)
Later than peers, n (%)	2514 (17.1)	87 (17.7)	88 (15.0)	2689 (17.1)
Missings, n (%)	117 (0.8)	4 (0.8)	1 (0.2)	122 (0.8)
Daily number of cigarettes in 1st trimester, n (%)				
Non-smoker, n (%)	10479 (71.3)	396 (80.3)	434 (74.2)	11309 (71.7)
−10 cigarettes/day, n (%)	3304 (22.5)	72 (14.6)	125 (21.4)	3501 (22.2)
>10 cigarettes/day, n (%)	857 (5.8)	23 (4.7)	26 (4.4)	906 (5.8)
Missings, n (%)	51 (0.4)	2 (0.4)	0 (0.0)	53 (0.3)
Alcohol units per week in 1st trimester, n (%)				
0 units, n (%)	7599 (51.7)	259 (52.5)	274 (46.8)	8132 (51.6)
>0–1 units, n (%)	4576 (31.2)	145 (29.4)	192 (32.8)	4913 (31.2)
>1–3 units, n (%)	1743 (11.9)	62 (12.6)	94 (16.1)	1899 (12.0)
>3 units, n (%)	752 (5.1)	26 (5.3)	25 (4.3)	803 (5.1)
Missings, n (%)	21 (0.1)	1 (0.2)	0 (0.0)	22 (0.1)
Highest social class of parents, n (%)				
High grade professional, n (%)	3360 (22.9)	141 (28.6)	175 (29.9)	3676 (23.3)
Low grade professional, n (%)	4823 (32.8)	161 (32.7)	192 (32.8)	5176 (32.8)
Skilled worker, n (%)	4111 (28.0)	110 (22.3)	127 (21.7)	4348 (27.6)
Unskilled worker, n (%)	2003 (13.6)	63 (12.8)	71 (12.1)	2137 (13.6)
Student, n (%)	284 (1.9)	10 (2.0)	16 (2.7)	310 (2.0)
Economically inactive, n (%)	81 (0.6)	6 (1.2)	4 (0.7)	91 (0.6)
Missings, n (%)	29 (0.2)	2 (0.4)	0 (0.0)	31 (0.2)
Asthma in pregnancy, n (%)				
No, n (%)	14059 (95.7)	176 (35.7)	518 (88.6)	14753 (93.6)
Yes, n (%)	628 (4.3)	317 (64.3)	67 (11.5)	1012 (6.4)
Missings, n (%)	4 (0.03)	0 (0.0)	0 (0.0)	4 (0.03)
Allergy in pregnancy, n (%)				
No, n (%)	12957 (88.2)	163 (33.1)	306 (52.3)	13426 (85.1)
Yes, n (%)	1692 (11.5)	328 (66.5)	277 (47.4)	2297 (14.6)
Missings, n (%)	42 (0.3)	2 (0.4)	2 (0.3)	46 (0.3)
Skin disease in pregnancy, n (%)				
No, n (%)	13889 (94.5)	431 (87.4)	315 (53.9)	14635 (92.8)
Yes, n (%)	749 (5.1)	59 (12.0)	267 (45.6)	1075 (6.8)
Missings, n (%)	53 (0.4)	3 (0.6)	3 (0.5)	59 (0.4)
Muscle or joint disease in pregnancy, n (%)				
No, n (%)	13000 (88.5)	422 (85.6)	494 (84.4)	13916 (88.3)
Yes, n (%)	1649 (11.2)	67 (13.6)	91 (15.6)	1807 (11.5)
Missings, n (%)	42 (0.3)	4 (0.8)	0 (0.0)	46 (0.3)

^1^ICS: inhaled corticosteroids. ^2^INCS1: first-generation intranasal corticosteroids.^3^Other include intranasal corticosteroids second-generation, cream, tablets, injections, suppositories, eye- and eardrops. ^4^BMI: Body-Mass-Index.

**Figure 2:**
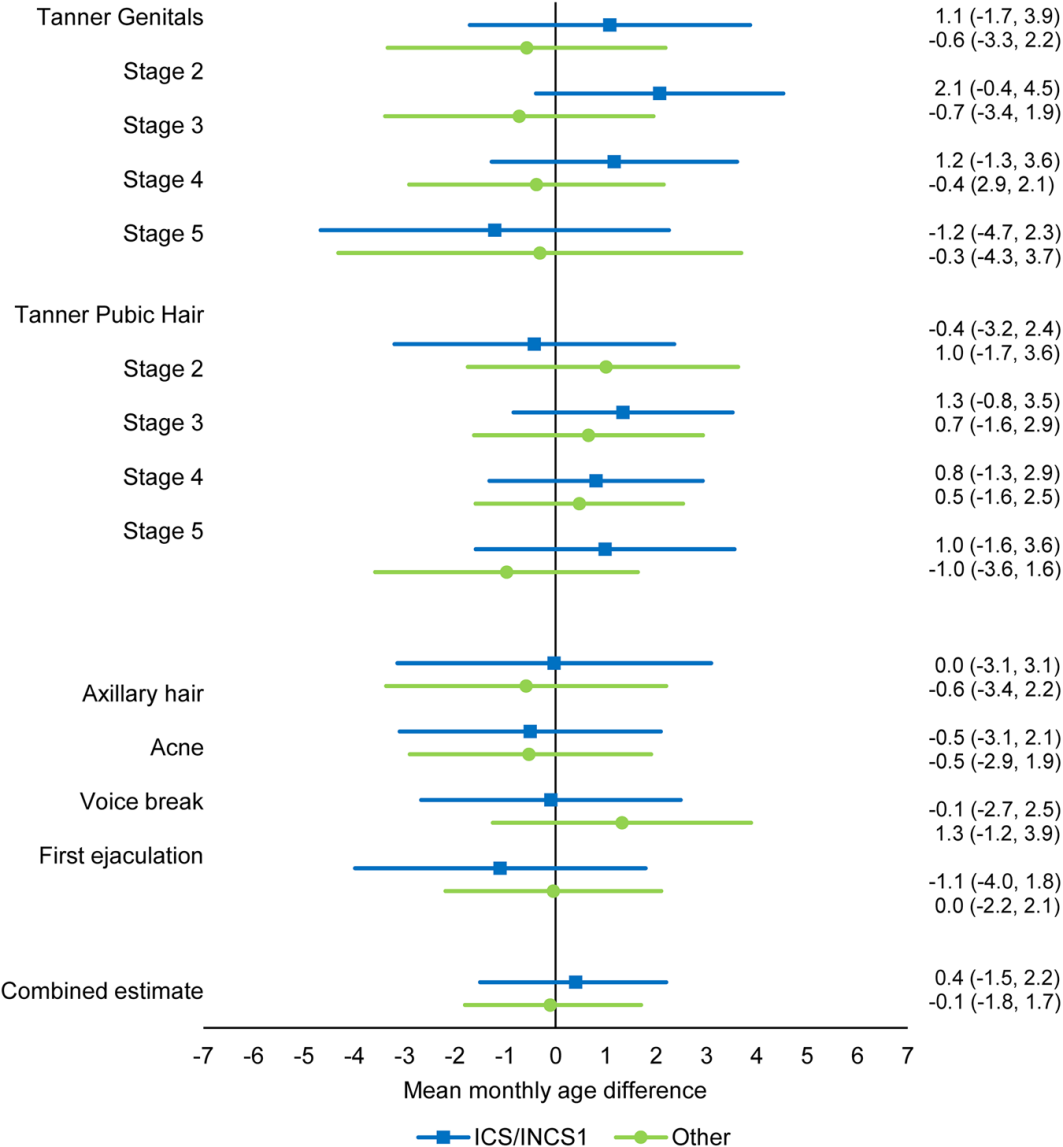
Adjusted mean monthly age differences in attaining different pubertal markers in boys across exposure groups according to pharmaceutical formulation with 95% CI. ICS: inhaled corticosteroids. INCS1: first-generation intranasal corticosteroids. Others include second-generation intranasal corticosteroids, cream, tablets, injections, suppositories, eye- and eardrops. N = 7,676.

**Figure 3:**
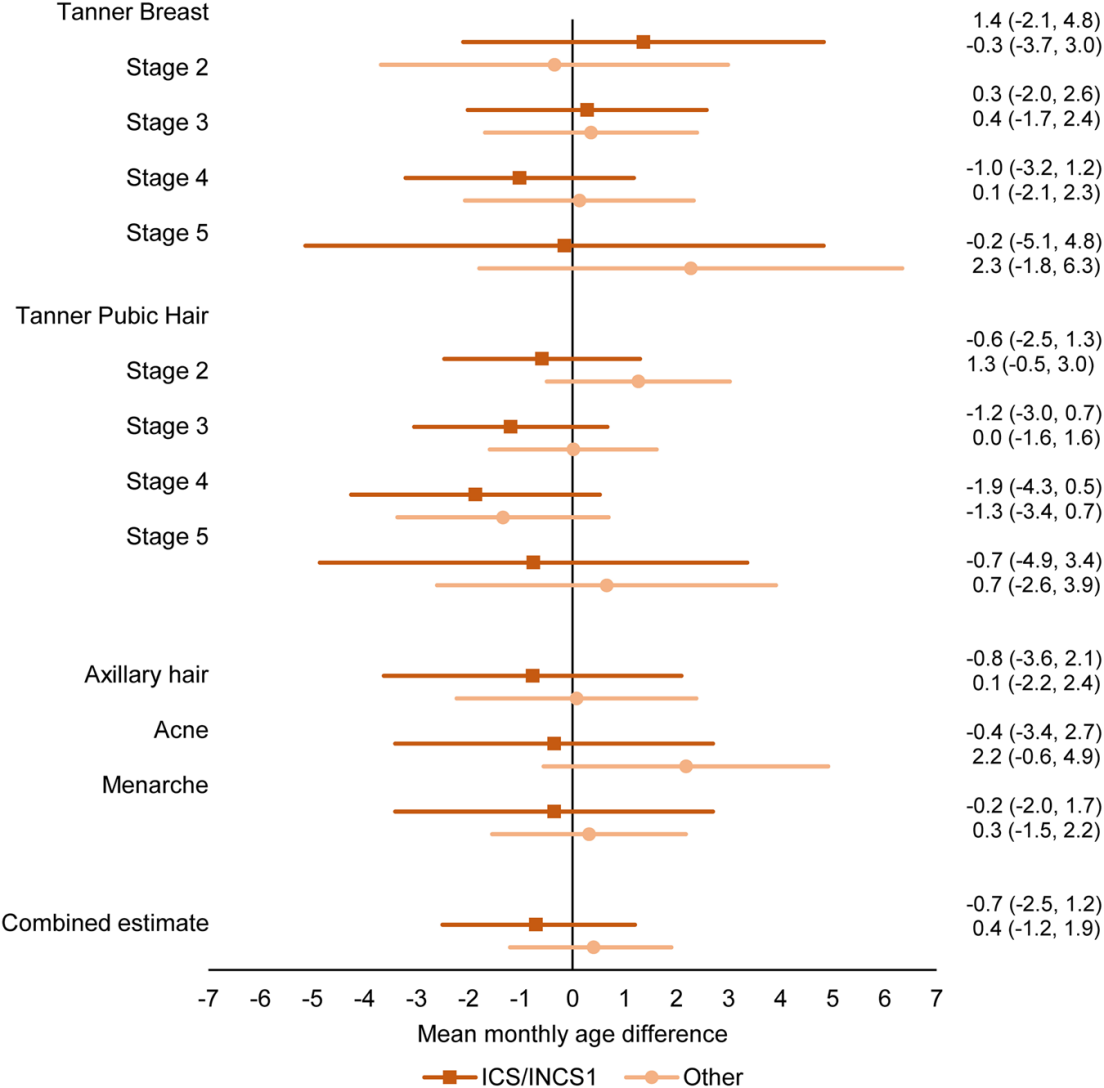
Adjusted mean monthly age differences in attaining different pubertal markers in girls across exposure groups according to pharmaceutical formulation with 95% CI. ICS: inhaled corticosteroids. INCS1: first-generation intranasal corticosteroids. Others include second-generation intranasal corticosteroids, cream, tablets, injections, suppositories, eye- and eardrops. N = 8,093.

We found no difference in age at attaining the different pubertal markers in the crude or the adjusted main analysis for neither boys nor girls (Figs. 2 and 3, Supplementary Table 1), and the estimates for the combined association between all pubertal markers did not show any difference in pubertal timing for boys (0.4 (95% confidence interval (CI): −1.5; 2.2) months) or girls (−0.7 (95% CI: −2.5; 1.2) months). No association between the duration of exposure and timing of puberty for neither boys nor girls was found (Supplementary Table 2). Excluding children born before gestational week 34 + 0 (Supplementary Table 3. Combined estimate boys: 0.4 (95% CI: −1.5; 2.2), combined estimate girls: −0.7 (95% CI: −2.6; 1.1)), adjusting for childhood asthma (Supplementary Table 4. Combined estimate boys: 0.5 (95% CI: −1.6; 2.6), combined estimate girls: −1.7 (95% CI: −3.8; 0.4)) or adjusting for childhood BMI (Supplementary Table 5. Combined estimate boys: 0.6 (95% CI: −1.5; 2.7), combined estimate girls: −1.7 (95% CI: −3.8; 0.4)) did not essentially change the results obtained in the main analysis.

**Figure 4:**
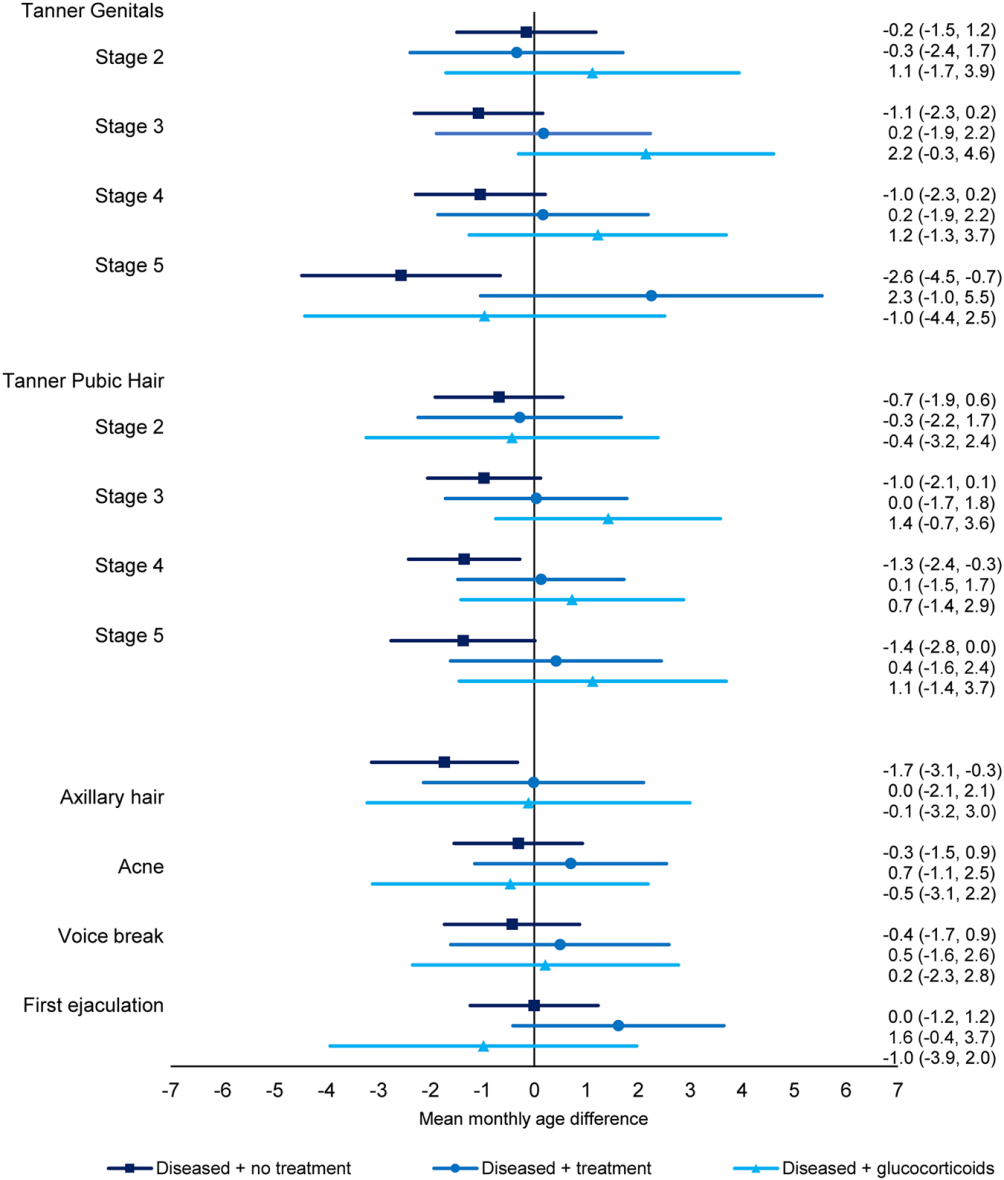
Adjusted mean monthly age differences in attaining different pubertal markers in boys across exposure groups according to disease and treatment status with 95% CI. Diseased refers to asthma, allergy, skin diseases or muscle and joint diseases, treatment refers to treatment for the disease without glucocorticoids. N = 7,297.

**Figure 5:**
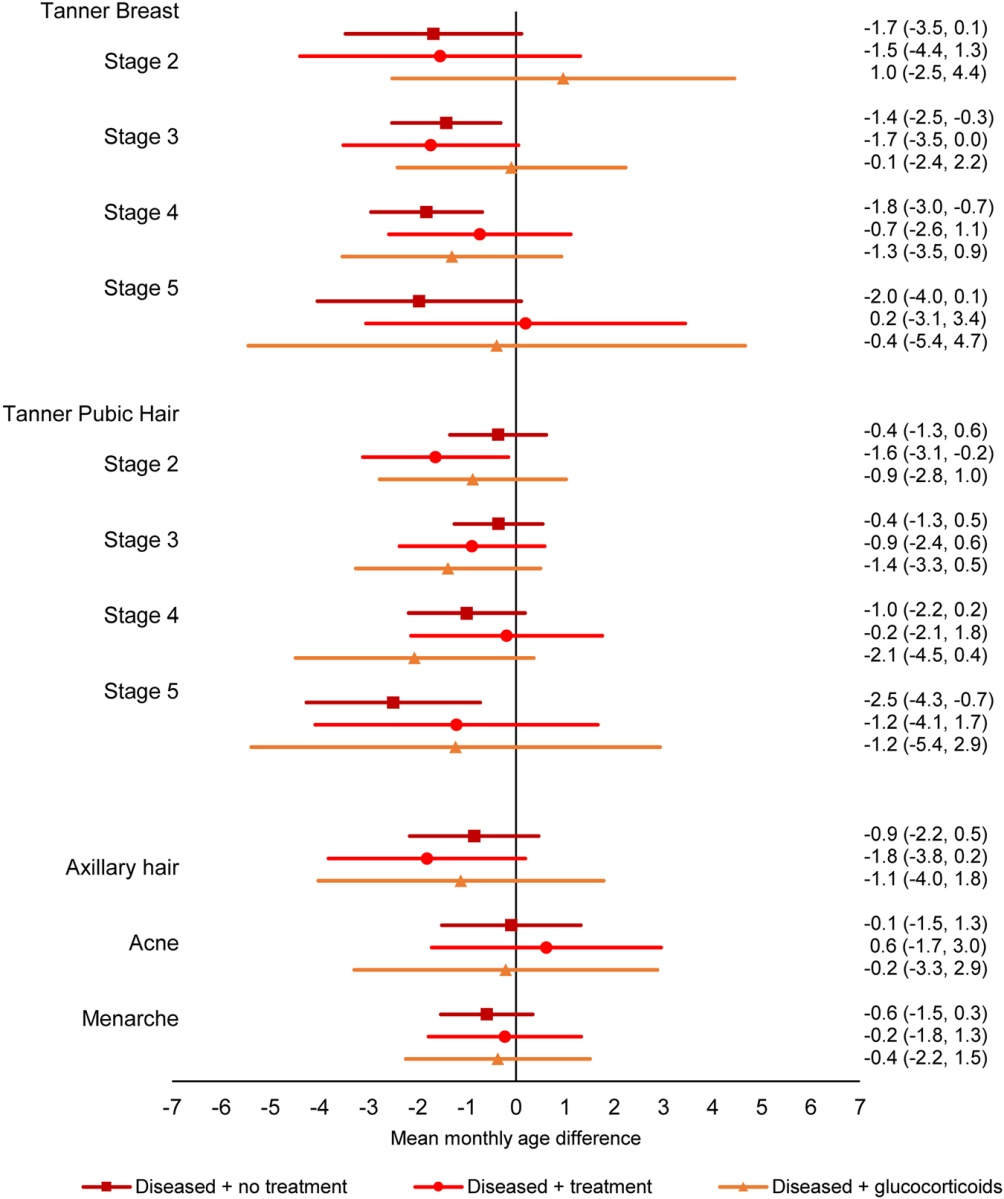
Adjusted mean monthly age differences in attaining different pubertal markers in girls across exposure groups according to disease and treatment status with 95% CI. Diseased refers to asthma, allergy, skin diseases or muscle and joint diseases, treatment refers to treatment for the disease without glucocorticoids. N = 7,693.

In the sub-analysis exploring confounding by indication, a tendency towards earlier pubertal timing was observed for sons and daughters of mothers who had one of the diseases of interest, but who did not receive treatment (Figs. 4 and 5, Supplementary Table 6, Combined estimate boys: −0.9 (95% CI: −1.7; 0.0), combined estimate girls: −1.1 (95% CI: −1.9; −0.2)). The association was most pronounced for girls in Tanner Breast stage 5 and Tanner Pubic Hair stage 5, with estimates of mean monthly age differences at −2.0 (95% CI: −4.0; 0.1) and −2.5 (95% CI: −4.3; −0.7). These associations moved towards the null for women treated for their disease (Combined estimate boys: 0.4 (95% CI: −0.9; 1.7), combined estimate girls: −0.9 (95% CI: −2.2; 0.4)), including treatment with glucocorticoids (Combined estimate boys: 0.5 (95% CI: −1.4; 2.3), combined estimate girls: −0.9 (95% CI: −2.8, 1.0)).

## Discussion

In this study, in utero exposure to glucocorticoids was not associated with pubertal timing across various pubertal markers for boys and girls, and no association between the duration of exposure and pubertal timing was observed. However, a tendency towards earlier pubertal timing was observed among sons and daughters of mothers reporting to have one of the diseases of interest (asthma, allergy, skin diseases and muscle or joint diseases) without being treated compared to children of mothers with a disease who received treatment. However, these differences were small and must be interpreted with caution.

No previous studies among humans have investigated the association between in utero exposure to glucocorticoids and pubertal timing in the children. However, one experimental rat study have suggested that exposure to glucocorticoids was associated with delayed puberty^31^. Animal studies may not be comparable with human studies due to differences in neurodevelopmental profiles across species^32^, no juvenile pause in rats and the study used a high dose of glucocorticoids which is different from our study. Previous human studies have examined the HPA-axis activity through basal cortisol levels and reactivity to stress after exposure to glucocorticoids in utero^10,33^. In general, these studies found decreased HPA-axis activity in infancy and focused on glucocorticoids given to pregnant women at risk of preterm birth, which are more potent than the glucocorticoids being evaluated in our study. Moreover, they are often given once or twice late in pregnancy rather than continuously throughout pregnancy, and, therefore, differences may be observed. However, few studies have also investigated the use of ICS^15^ and INCS^34^. A large study within the DNBC found an association between in utero exposure to ICS and metabolic and endocrine diseases in children around age 6 years^15^.

Strengths of the study include the large study population and information on various pubertal markers with continuous follow-up for boys and girls. Both information on treatment and puberty was self-reported, which introduces the risk of misclassification. However, interviews and questionnaires were conducted near the event, which reduces the risk of recall bias. A validation study within the DNBC conclude that women are more likely to report medicine for chronic diseases rather than medicine for local use or short-term treatment, which implies that women treated for e.g. asthma may be more likely to report use of medication compared to women treated with local corticosteroids for a skin disease^35^. Due to the risk of underreporting, it cannot be ruled out that women in the untreated group may be truly treated, and, thereby, the results would be biased towards the null. Some women may also have been treated with glucocorticoids due to risk of preterm birth, but since exclusion of children born before week 34 + 0 did not change the results, we consider this to be of minor importance in the study. However, we cannot exclude that there may be women who were treated with glucocorticoids and following giving birth to a full-term baby, but we consider this proportion to be limited.

Another validation study in the Puberty Cohort^36^ showed some misclassification on the self-reported pubertal staging compared to clinical assessment, but the estimated agreements were reported as fair to moderate indicating that self-reported staging is still a useful tool. Self-reported puberty staging is time and cost saving with high participation rates, and gives the opportunity to analyse large cohorts which is needed especially for boys within research on pubertal timing. The potential misclassification in this study is expected to be non-differential given that information on exposure and outcome was obtained independently of each other, as pregnant women were not aware of their unborn child’s future pubertal timing, and the children would not be prone to answer questions on pubertal stages according to their mothers’ treatment with glucocorticoids.

Glucocorticoids of various formulations differ in systemic absorption, for example tablets^37^ versus cream^38^, and may therefore not have the same effect on pubertal timing. We primarily focused on the group exposed to ICS and first-generation INCS, and one should be careful when concluding upon the results for the other formulations. The association between duration of treatment and pubertal timing was explored by using information on which gestational weeks the women were treated. No associations were found in this analysis, which may be due to lack of exposure contrast, as a large proportion (49%) had been treated every gestational week until week 28. The number of treated weeks was used as a surrogate marker for the treatment dose. It may also be relevant to consider timing of treatment, as the fetus may be more vulnerable to exposures during the first trimester^39^. The data used in this study concerns the first two trimesters, and the majority have been exposed every week; therefore, a trimester specific analysis was not performed.

Although the study has a relatively high participation rate, about 70%, risk of selection bias must be considered. Glucocorticoid exposure was similar between participants (6.8%) and non-participants (5.5%). Therefore, we do not expect that selection bias has biased our findings. Still, selection weights were included in the analyses to reduce the risk of selection bias.

It is challenging to separate the effect of the medicine from the disease itself, but results from our sub-analysis showed that the disease itself seems to be associated with earlier puberty for both boys and girls, rather than the use of glucocorticoids. There might be different explanations for this. First, untreated disease may be associated with earlier puberty. Second, diseases treated with glucocorticoids showed no clear effect on pubertal timing, which may be due to glucocorticoids delaying pubertal timing and, thereby, cancel out the effect of the disease. This potential explanation is contradictory to the initial hypothesis, which suggests that glucocorticoids accelerate pubertal timing. However, from this study we are not able to rule out that other unknown biological mechanisms may be at play. Third, the results can be chance findings. Either way, the results must be interpreted with caution as the estimated differences were small. In general, studies find it safer to use ICS for asthma, rather than refusing treatment during pregnancy, when evaluating health implications for the child^40^. This recommendation was supported by the results for pubertal timing in the children obtained in this study.

In conclusion, in utero exposure to glucocorticoids was not associated with pubertal timing in the children. No association between duration of treatment with glucocorticoids and pubertal timing was observed. The results are reassuring for women who suffer from diseases where glucocorticoids are indicated.

## Supplementary information

Supplementary Information

## Data Availability

The dataset analysed in the study is not publicly available due to national data security legislation on sensitive personal data.
