# Supplementary material for: In Utero Exposure to Glucocorticoids and Pubertal Timing in Sons and Daughters
Source: Sci Rep. 2019 Dec 30;9:20374. doi: 10.1038/s41598-019-56917-7 (PMC6937234; doi:10.1038/s41598-019-56917-7)
Supplement: Supplementary file 1 — Supplementary Information [file 41598_2019_56917_MOESM1_ESM.pdf]

Supplementary material

In Utero Exposure to Glucocorticoids and Pubertal Timing in Sons and Daughters

Scientific Reports

Sofie Aagaard Sand, MB[1], Andreas Ernst, MD, PhD[1,2], Lea Lykke Harrits Lunddorf, MD[1], Nis Brix, MD[1], Anne Gaml-Sørensen, MHSc[1], Cecilia Høst Ramlau-Hansen, MHSc, PhD[1]

[1]Department of Public Health, Research Unit for Epidemiology, Aarhus University, Aarhus, Denmark

[2]Department of Urology, Aarhus University Hospital, Aarhus, Denmark

Corresponding author

Sofie Aagaard Sand

sofie.sand@outlook.com

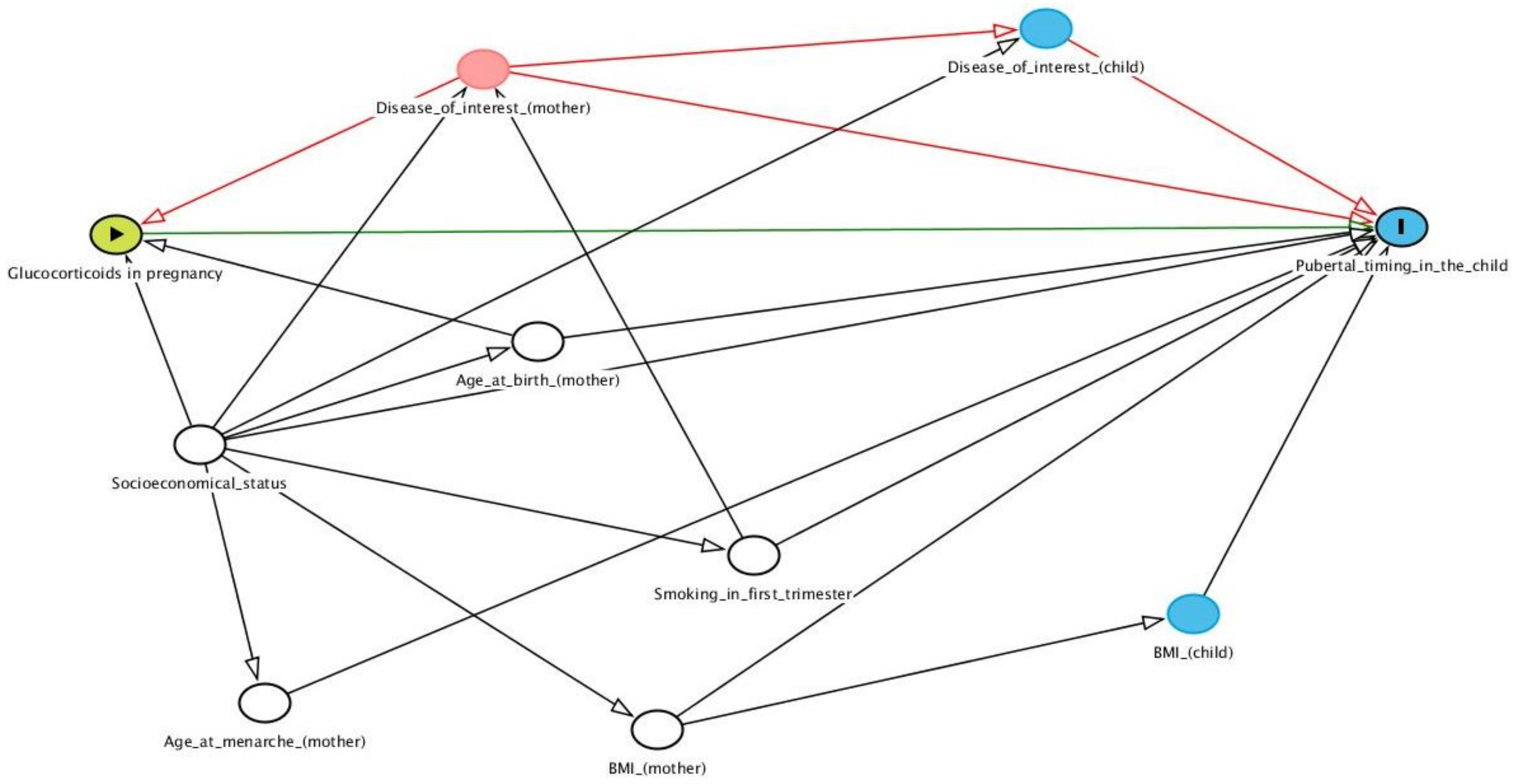

Supplementary Fig. 1 Directed acyclic graph used to identify potential confounders

Supplementary Table 1 Mean monthly age differences in pubertal timing according to in utero glucocorticoid exposure in the Puberty Cohort

			Exposure to Glucocorticoids								
			Unexposed		ICS/INCS1			Other			
	Pubertal Milestones	No.	Mean Age	95 % CI	Crude Mean Diff.	Adjusted Mean Diff.	95 % CI	Crude Mean Diff.	Adjusted Mean Diff.	95 % CI
Boys	Tanner Genitals									
	Stage 2	7458	10.6	10.2, 10.9	1.2	1.1	-1.7, 3.9	-0.5	-0.6	-3.3, 2.2
	Stage 3	7458	12.0	11.7, 12.4	2.2	2.1	-0.4, 4.5	-0.7	-0.7	-3.4, 1.9
	Stage 4	7458	13.2	12.8, 13.6	1.1	1.2	-1.3, 3.6	-0.4	-0.4	-2.9, 2.1
	Stage 5	7458	15.3	14.7, 15.9	-1.3	-1.2	-4.7, 2.3	-0.3	-0.3	-4.3, 3.7
	Tanner Pubic Hair									
	Stage 2	7462	11.0	10.7, 11.3	-0.3	-0.4	-3.2, 2.4	0.9	1.0	-1.7, 3.6
	Stage 3	7462	12.3	12.0, 12.7	1.3	1.3	-0.8, 3.5	0.7	0.7	-1.6, 2.9
	Stage 4	7462	13.2	12.9, 13.6	0.7	0.8	-1.3, 2.9	0.5	0.5	-1.6, 2.5
	Stage 5	7462	14.5	14.0, 14.9	0.9	1.0	-1.6, 3.6	-0.9	-1.0	-3.6, 1.6
	Axillary hair	7467	13.4	13.0, 13.9	-0.1	0.0	-3.1, 3.1	-0.4	-0.6	-3.4, 2.2
	Acne	7467	11.7	11.3, 12.0	-0.5	-0.5	-3.1, 2.1	-0.3	-0.5	-2.9, 1.9
	Voice break	7264	12.6	12.3, 13.0	0.1	-0.1	-2.7, 2.5	1.4	1.3	-1.2, 3.9
	First ejaculation	7454	13.0	12.6, 13.4	-1.2	-1.1	-4.0, 1.8	-0.2	0.0	-2.2, 2.1
	Combined estimate	7264				0.4	-1.5, 2.2		-0.1	-1.8, 1.7
Girls	Tanner Breast									
	Stage 2	7869	9.5	8.9, 10.1	1.1	1.4	-2.1, 4.8	-0.8	-0.4	-3.7, 3.0
	Stage 3	7869	11.1	10.7, 11.4	-0.2	0.3	-2.0, 2.6	-0.2	0.4	-1.7, 2.4
	Stage 4	7869	12.8	12.4, 13.1	-1.4	-1.0	-3.2, 1.2	-0.3	0.1	-2.1, 2.3
	Stage 5	7869	16.1	15.5, 16.8	-0.5	-0.2	-5.1, 4.8	2.0	2.3	-1.8, 6.3
	Tanner Pubic Hair									
	Stage 2	7870	10.9	10.6, 11.2	-0.9	-0.6	-2.5, 1.3	0.9	1.3	-0.5, 3.0
	Stage 3	7870	12.1	11.8, 12.4	-1.6	-1.2	-3.0, 0.7	-0.4	0.0	-1.6, 1.6
	Stage 4	7870	13.3	12.9, 13.7	-2.2	-1.9	-4.3, 0.5	-1.8	-1.3	-3.4, 0.7
	Stage 5	7870	16.1	15.5, 16.6	-0.8	-0.7	-4.9, 3.4	0.2	0.7	-2.6, 3.9
	Axillary hair	7875	11.6	11.2, 12.0	-1.3	-0.8	-3.6, 2.1	-0.5	0.1	-2.2, 2.4
	Acne	7875	11.2	10.7, 11.7	-0.6	-0.4	-3.4, 2.7	1.7	2.2	-0.6, 4.9
	Menarche	7867	12.7	12.4, 13.0	-0.4	-0.2	-2.0, 1.7	0.0	0.3	-1.5, 2.2
	Combined estimate	7867				-0.7	-2.5, 1.2		0.4	-1.2, 1.9

ICS: inhaled corticosteroids. INCS1: first-generation intranasal corticosteroids. Others include second-generation intranasal corticosteroids, cream, tablets, injections, suppositories, eye- and eardrops.

Supplementary Table 2 Mean monthly age differences in pubertal timing according to in utero glucocorticoid exposure and duration of exposure in the Puberty Cohort

			Exposure to Glucocorticoids (ICS/INCS1)				
			Unexposed		Exposed		
	Pubertal Milestones	No.	Mean Age	95 % CI	Crude Mean Diff.	Adjusted Mean Diff.	95 % CI
Boys	Tanner Genitals						
	Stage 2	7186	10.6	10.3, 11.0	0.3	0.3	-0.4, 1.1
	Stage 3	7186	12.1	11.7, 12.4	0.5	0.5	-0.1, 1.0
	Stage 4	7186	13.2	12.8, 13.6	0.3	0.4	-0.2, 0.9
	Stage 5	7186	15.3	14.7, 15.9	0.3	0.3	-0.5, 1.2
	Tanner Pubic Hair						
	Stage 2	7189	11.0	10.7, 11.3	0.0	0.0	-0.7, 0.7
	Stage 3	7189	12.3	12.0, 12.7	0.2	0.3	-0.3, 0.8
	Stage 4	7189	13.2	12.9, 13.5	0.3	0.3	-0.2, 0.9
	Stage 5	7189	14.4	14.0, 14.9	0.4	0.5	-0.2, 1.2
	Axillary hair	7193	13.4	12.9, 13.8	0.0	0.1	-0.7, 0.9
	Acne	7193	11.6	11.3, 12.0	-0.2	-0.2	-0.8, 0.5
	Voice break	7002	12.6	12.2, 13.0	0.1	0.2	-0.4, 0.7
	First ejaculation	7182	13.0	12.6, 13.4	0.0	0.0	-0.7, 0.7
Girls	Tanner Breast						
	Stage 2	7569	9.5	8.9, 10.1	0.4	0.6	-0.3, 1.4
	Stage 3	7569	11.1	10.7, 11.5	0.1	0.3	-0.3, 0.8
	Stage 4	7569	12.7	12.3, 13.1	-0.1	0.0	-0.6, 0.6
	Stage 5	7569	16.0	15.3, 16.7	0.1	0.2	-1.0, 1.4
	Tanner Pubic Hair						
	Stage 2	7570	11.0	10.6, 11.3	-0.1	0.0	-0.5, 0.5
	Stage 3	7570	12.2	11.8, 12.5	-0.3	-0.2	-0.7, 0.2
	Stage 4	7570	13.3	12.9, 13.7	-0.3	-0.2	-0.9, 0.4
	Stage 5	7570	16.1	15.5, 16.6	0.2	0.3	-0.8, 1.4
	Axillary hair	7575	11.7	11.2, 12.1	-0.4	-0.3	-0.9, 0.4
	Acne	7575	11.2	10.8, 11.7	0.0	0.1	-0.6, 0.9
	Menarche	7567	12.7	12.4, 13.0	0.0	0.1	-0.4, 0.5

ICS: inhaled corticosteroids. INCS1: first-generation intranasal corticosteroids.

Supplementary Table 3 Mean monthly age differences in pubertal timing according to in utero glucocorticoid exposure when excluding children born before week 34+0 in the Puberty Cohort

			Exposure to Glucocorticoids							
			Unexposed		ICS/INCS1			Other		
					Crude	Adjusted		Crude	Adjusted	
	Pubertal Milestones	No.	Mean Age	95 % CI	Mean Diff.	Mean Diff.	95 % CI	Mean Diff.	Mean Diff.	95 % CI
Boys	Tanner Genitals									
	Stage 2	7331	10.6	10.2, 10.9	1.2	1.1	-1.7, 3.9	-0.6	-0.6	-3.4, 2.2
	Stage 3	7331	12.0	11.7, 12.4	2.2	2.0	-0.4, 4.5	-0.7	-0.7	-3.4, 2.0
	Stage 4	7331	13.2	12.8, 13.6	1.1	1.1	-1.3, 3.6	-0.3	-0.4	-2.9, 2.2
	Stage 5	7331	15.3	14.7, 15.9	-1.3	-1.2	-4.6, 2.3	-0.3	-0.3	-4.3, 3.7
	Tanner Pubic Hair									
	Stage 2	7335	11.0	10.7, 11.3	-0.4	-0.5	-3.3, 2.3	0.9	0.9	-1.8, 3.6
	Stage 3	7335	12.3	12.0, 12.7	1.3	1.3	-0.9, 3.5	0.7	0.6	-1.7, 2.9
	Stage 4	7335	13.2	12.9, 13.5	0.6	0.8	-1.4, 2.9	0.5	0.5	-1.6, 2.5
	Stage 5	7335	14.5	14.0, 14.9	0.9	1.0	-1.6, 3.5	-1.0	-1.0	-3.6, 1.6
	Axillary hair	7340	13.4	13.0, 13.9	-0.1	-0.1	-3.2, 3.1	-0.3	-0.5	-3.3, 2.3
	Acne	7340	11.7	11.3, 12.0	-0.6	-0.6	-3.2, 2.0	-0.3	-0.4	-2.8, 1.9
	Voice break	7138	12.6	12.3, 13.0	0.1	-0.1	-2.7, 2.5	1.7	1.6	-1.0, 4.1
	First ejaculation	7327	12.9	12.5, 13.3	-1.1	-1.0	-3.9, 1.9	-0.2	0.0	-2.2, 2.1
	Combined estimate	7138				0.4	-1.5, 2.2		0.0	-1.8, 1.8
Girls	Tanner Breast									
	Stage 2	7764	9.5	8.9, 10.1	0.9	1.2	-2.3, 4.8	-0.8	-0.3	-3.7, 3.0
	Stage 3	7764	11.1	10.7, 11.4	-0.2	0.2	-2.1, 2.5	-0.2	0.3	-1.7, 2.4
	Stage 4	7764	12.8	12.4, 13.1	-1.4	-1.0	-3.2, 1.2	-0.3	0.1	-2.1, 2.3
	Stage 5	7764	16.2	15.5, 16.8	-0.6	-0.2	-5.2, 4.8	2.1	2.3	-1.7, 6.4
	Tanner Pubic Hair									
	Stage 2	7765	10.9	10.6, 11.2	-1.0	-0.7	-2.6, 1.2	0.9	1.3	-0.5, 3.0
	Stage 3	7765	12.1	11.8, 12.4	-1.7	-1.2	-3.1, 0.6	-0.4	0.0	-1.6, 1.6
	Stage 4	7765	13.3	12.9, 13.6	-2.3	-1.9	-4.3, 0.5	-1.8	-1.4	-3.4, 0.7
	Stage 5	7765	16.1	15.5, 16.6	-0.9	-0.8	-4.9, 3.3	0.1	0.6	-2.7, 3.9
	Axillary hair	7770	11.6	11.2, 12.0	-1.4	-0.9	-3.7, 2.0	-0.5	0.0	-2.3, 2.3
	Acne	7770	11.2	10.7, 11.7	-0.7	-0.5	-3.6, 2.6	1.7	2.2	-0.6, 4.9
	Menarche	7762	12.7	12.4, 13.0	-0.4	-0.2	-2.1, 1.6	-0.1	0.3	-1.6, 2.2
	Combined estimate	7762				-0.7	-2.6, 1.1		0.4	-1.2, 1.9

ICS: inhaled corticosteroids. INCS1: first-generation intranasal corticosteroids. Others include second-generation intranasal corticosteroids, cream, tablets, injections, suppositories, eye- and eardrops.

Supplementary Table 4 Mean monthly age differences in pubertal timing according to in utero glucocorticoid exposure additionally adjusted for childhood asthma in the Puberty Cohort

			Exposure to Glucocorticoids							
			Unexposed		ICS/INCS1			Other		
					Crude	Adjusted		Crude	Adjusted	
	Pubertal Milestones	No.	Mean Age	95 % CI	Mean Diff.	Mean Diff.	95 % CI	Mean Diff.	Mean Diff.	95 % CI
Boys	Tanner Genitals									
	Stage 2	5466	10.4	9.9, 10.9	1.2	1.6	-1.5, 4.7	-0.5	0.9	-2.1, 3.9
	Stage 3	5466	11.7	11.2, 12.2	2.2	2.0	-0.9, 4.8	-0.7	0.5	-2.6, 3.5
	Stage 4	5466	13.1	12.6, 13.5	1.1	1.0	-1.8, 3.8	-0.4	0.0	-2.9, 2.8
	Stage 5	5466	14.9	14.1, 15.7	-1.3	-0.5	-4.4, 3.4	-0.3	0.8	-3.8, 5.5
	Tanner Pubic Hair									
	Stage 2	5467	10.8	10.4, 11.2	-0.3	-0.1	-3.2, 3.0	0.9	2.5	-0.5, 5.6
	Stage 3	5467	12.2	11.7, 12.6	1.3	1.8	-0.7, 4.2	0.7	2.1	-0.5, 4.6
	Stage 4	5467	13.1	12.7, 13.5	0.7	0.6	-1.7, 3.0	0.5	0.9	-1.5, 3.3
	Stage 5	5467	14.3	13.7, 14.9	0.9	2.1	-0.8, 5.0	-0.9	-0.1	-3.1, 2.8
	Axillary hair	5470	13.6	13.0, 14.1	-0.1	-0.3	-3.8, 3.3	-0.4	-0.5	-3.7, 2.8
	Acne	5470	11.7	11.2, 12.1	-0.5	-0.8	-3.8, 2.1	-0.3	-0.5	-3.2, 2.3
	Voice break	5358	12.4	12.0, 12.9	0.1	-0.8	-3.6, 2.1	1.4	1.9	-1.1, 4.8
	First ejaculation	5463	12.9	12.4, 13.4	-1.2	-1.2	-4.5, 2.0	-0.2	0.3	-2.3, 2.8
	Combined estimate	5358				0.5	-1.6, 2.6		0.7	-1.4, 2.7
Girls	Tanner Breast									
	Stage 2	5588	9.7	9.0, 10.4	1.1	-0.4	-4.3, 3.6	-0.8	-0.3	-4.1, 3.5
	Stage 3	5588	11.2	10.8, 11.6	-0.2	-0.7	-3.4, 2.1	-0.2	-0.3	-2.8, 2.2
	Stage 4	5588	12.8	12.4, 13.2	-1.4	-1.9	-4.3, 0.6	-0.3	0.0	-2.5, 2.6
	Stage 5	5588	15.9	15.2, 16.7	-0.5	-2.1	-7.8, 3.5	2.0	1.8	-3.0, 6.6
	Tanner Pubic Hair									
	Stage 2	5588	11.0	10.7, 11.3	-0.9	-1.1	-3.1, 1.0	0.9	-0.7	-2.7, 1.3
	Stage 3	5588	12.2	11.9, 12.6	-1.6	-2.0	-4.0, 0.0	-0.4	-0.5	-2.3, 1.3
	Stage 4	5588	13.4	13.0, 13.8	-2.2	-3.2	-6.0, -0.4	-1.8	-1.6	-4.1, 0.8
	Stage 5	5588	16.2	15.6, 16.8	-0.8	-2.3	-6.7, 2.1	0.2	0.8	-3.0, 4.6
	Axillary hair	5591	11.8	11.3, 12.3	-1.3	-1.8	-5.2, 1.7	-0.5	0.0	-2.8, 2.8
	Acne	5591	11.2	10.7, 11.7	-0.6	-1.6	-5.2, 1.9	1.7	1.8	-1.5, 5.1
	Menarche	5586	12.7	12.3, 13.0	-0.4	-0.9	-2.9, 1.2	0.0	-0.6	-2.8, 1.6
	Combined estimate	5586				-1.7	-3.8, 0.4		-0.2	-2.0, 1.7

ICS: inhaled corticosteroids. INCS1: first-generation intranasal corticosteroids. Others include second-generation intranasal corticosteroids, cream, tablets, injections, suppositories, eye- and eardrops.

Supplementary Table 5 Mean monthly age differences in pubertal timing according to in utero glucocorticoid exposure additionally adjusted for childhood BMI in the Puberty Cohort

			Exposure to Glucocorticoids							
			Unexposed		ICS/INCS1			Other		
					Crude	Adjusted		Crude	Adjusted	
	Pubertal Milestones	No.	Mean Age	95 % CI	Mean Diff.	Mean Diff.	95 % CI	Mean Diff.	Mean Diff.	95 % CI
Boys	Tanner Genitals									
	Stage 2	5333	11.1	10.4, 11.8	1.2	1.9	-1.3, 5.1	-0.5	0.8	-2.2, 3.9
	Stage 3	5333	12.9	12.3, 13.6	2.2	2.2	-0.6, 5.1	-0.7	-0.1	-3.1, 2.9
	Stage 4	5333	13.6	12.8, 14.3	1.1	0.9	-1.9, 3.7	-0.4	-0.4	-3.2, 2.4
	Stage 5	5333	15.5	14.6, 16.4	-1.3	-0.7	-4.5, 3.2	-0.3	0.5	-4.2, 5.1
	Tanner Pubic Hair									
	Stage 2	5334	11.1	10.5, 11.7	-0.3	0.1	-3.0, 3.3	0.9	1.8	-1.1, 4.7
	Stage 3	5334	12.8	11.9, 13.6	1.3	2.0	-0.5, 4.5	0.7	1.7	-0.8, 4.2
	Stage 4	5334	13.6	12.9, 14.3	0.7	0.8	-1.6, 3.2	0.5	0.5	-1.9, 2.8
	Stage 5	5334	15.0	14.1, 15.9	0.9	2.0	-0.9, 4.8	-0.9	-0.3	-3.3, 2.6
	Axillary hair	5336	14.6	13.3, 15.8	-0.1	-0.2	-3.8, 3.3	-0.4	-1.0	-4.2, 2.1
	Acne	5336	12.1	11.4, 12.9	-0.5	-0.4	-3.3, 2.6	-0.3	-0.9	-3.6, 1.8
	Voice break	5232	13.1	12.3, 13.9	0.1	-0.5	-3.3, 2.4	1.4	1.2	-1.8, 4.2
	First ejaculation	5330	13.3	12.7, 13.9	-1.2	-1.2	-4.5, 2.0	-0.2	-0.2	-2.8, 2.3
	Combined estimate	5232				0.6	-1.5, 2.7		0.2	-1.8, 2.2
Girls	Tanner Breast									
	Stage 2	5411	13.0	12.0, 14.0	1.1	-0.4	-4.2, 3.4	-0.8	-0.5	-4.4, 3.5
	Stage 3	5411	12.1	11.1, 13.1	-0.2	-0.5	-3.2, 2.2	-0.2	-0.6	-3.1, 2.0
	Stage 4	5411	13.6	12.8, 14.5	-1.4	-1.5	-4.0, 0.9	-0.3	-0.3	-2.9, 2.3
	Stage 5	5411	17.1	15.9, 18.4	-0.5	-2.2	-7.8, 3.5	2.0	1.2	-3.5, 5.9
	Tanner Pubic Hair									
	Stage 2	5411	11.8	11.3, 12.4	-0.9	-1.2	-3.3, 0.9	0.9	-0.8	-2.8, 1.2
	Stage 3	5411	12.8	12.2, 13.4	-1.6	-2.1	-4.1, -0.1	-0.4	-0.7	-2.5, 1.0
	Stage 4	5411	14.1	13.4, 14.8	-2.2	-3.5	-6.3, -0.7	-1.8	-1.9	-4.3, 0.6
	Stage 5	5411	17.2	16.2, 18.2	-0.8	-2.8	-7.1, 1.6	0.2	0.0	-3.7, 3.7
	Axillary hair	5414	12.6	11.7, 13.5	-1.3	-1.5	-5.0, 1.9	-0.5	-0.4	-3.2, 2.3
	Acne	5414	11.3	10.6, 12.1	-0.6	-1.7	-5.2, 1.9	1.7	1.5	-1.9, 4.8
	Menarche	5409	13.5	12.7, 14.3	-0.4	-0.8	-2.9, 1.3	0.0	-0.6	-2.8, 1.6
	Combined estimate	5409				-1.7	-3.8, 0.4		-0.4	-2.3, 1.4

ICS: inhaled corticosteroids. INCS1: first-generation intranasal corticosteroids. Others include second-generation intranasal corticosteroids, cream, tablets, injections, suppositories, eye- and eardrops.

Supplementary Table 6 Mean monthly age differences in pubertal timing according to in utero disease and medication status in the Puberty Cohort

			Exposure to Glucocorticoids (ICS/INCS1)										
			Unexposed		Group 2 (diseased + no treatment)			Group 3 (diseased + treatment)			Group 4 (diseased + glucocorticoids)		
	Pubertal Milestones	No.	Mean Age	95 % CI	Crude Mean Diff.	Adjusted Mean Diff.	95 % CI	Crude Mean Diff.	Adjusted Mean Diff.	95 % CI	Crude Mean Diff.	Adjusted Mean Diff.	95 % CI
Boys	Tanner Genitals												
	Stage 2	7091	10.6	10.3, 11.0	-0.2	-0.2	-1.5, 1.2	-0.4	-0.3	-2.4, 1.7	1.2	1.1	-1.7, 3.9
	Stage 3	7091	12.1	11.7, 12.4	-1.3	-1.1	-2.3, 0.2	0.0	0.2	-1.9, 2.2	2.3	2.2	-0.3, 4.6
	Stage 4	7091	13.2	12.8, 13.6	-1.3	-1.0	-2.3, 0.2	0.0	0.2	-1.9, 2.2	1.1	1.2	-1.3, 3.7
	Stage 5	7091	15.3	14.7, 15.9	-2.9	-2.6	-4.5, -0.7	2.1	2.3	-1.0, 5.5	-1.2	-1.0	-4.4, 2.5
	Tanner Pubic Hair												
	Stage 2	7094	11.0	10.7, 11.3	-0.8	-0.7	-1.9, 0.6	-0.4	-0.3	-2.2, 1.7	-0.4	-0.4	-3.2, 2.4
	Stage 3	7094	12.3	12.0, 12.7	-1.3	-1.0	-2.1, 0.1	-0.2	0.0	-1.7, 1.8	1.3	1.4	-0.7, 3.6
	Stage 4	7094	13.2	12.9, 13.6	-1.7	-1.3	-2.4, -0.3	-0.1	0.1	-1.5, 1.7	0.5	0.7	-1.4, 2.9
	Stage 5	7094	14.4	14.0, 14.9	-1.8	-1.4	-2.8, 0.0	0.1	0.4	-1.6, 2.4	1.0	1.1	-1.4, 3.7
	Axillary hair	7098	13.4	13.0, 13.9	-2.2	-1.7	-3.1, -0.3	-0.5	0.0	-2.1, 2.1	-0.3	-0.1	-3.2, 3.0
	Acne	7098	11.6	11.3, 12.0	-0.7	-0.3	-1.5, 0.9	0.4	0.7	-1.1, 2.5	-0.6	-0.5	-3.1, 2.2
	Voice break	6910	12.6	12.2, 13.0	-0.8	-0.4	-1.7, 0.9	0.2	0.5	-1.6, 2.6	0.3	0.2	-2.3, 2.8
	First ejaculation	7087	13.0	12.6, 13.4	-0.2	0.0	-1.2, 1.2	1.5	1.6	-0.4, 3.7	-1.1	-1.0	-3.9, 2.0
	Combined estimate	6910				-0.9	-1.7, 0.0		0.4	-0.9, 1.7		0.5	-1.4, 2.3
Girls	Tanner Breast												
	Stage 2	7483	9.5	8.9, 10.2	-2.3	-1.7	-3.5, 0.1	-2.3	-1.5	-4.4, 1.3	0.6	1.0	-2.5, 4.4
	Stage 3	7483	11.2	10.8, 11.5	-1.9	-1.4	-2.5, -0.3	-2.2	-1.7	-3.5, 0.0	-0.7	-0.1	-2.4, 2.2
	Stage 4	7483	12.7	12.4, 13.1	-2.2	-1.8	-3.0, -0.7	-1.2	-0.7	-2.6, 1.1	-1.8	-1.3	-3.5, 0.9
	Stage 5	7483	16.0	15.3, 16.7	-2.6	-2.0	-4.0, 0.1	-0.3	0.2	-3.1, 3.4	-0.9	-0.4	-5.4, 4.7
	Tanner Pubic Hair												
	Stage 2	7484	11.0	10.7, 11.3	-0.5	-0.4	-1.3, 0.6	-1.8	-1.6	-3.1, -0.2	-1.3	-0.9	-2.8, 1.0
	Stage 3	7484	12.2	11.9, 12.5	-0.6	-0.4	-1.3, 0.5	-1.2	-0.9	-2.4, 0.6	-1.8	-1.4	-3.3, 0.5
	Stage 4	7484	13.3	12.9, 13.7	-1.2	-1.0	-2.2, 0.2	-0.5	-0.2	-2.1, 1.8	-2.5	-2.1	-4.5, 0.4
	Stage 5	7484	16.1	15.6, 16.7	-2.8	-2.5	-4.3, -0.7	-1.5	-1.2	-4.1, 1.7	-1.4	-1.2	-5.4, 2.9
	Axillary hair	7489	11.7	11.3, 12.1	-1.1	-0.9	-2.2, 0.5	-2.2	-1.8	-3.8, 0.2	-1.7	-1.1	-4.0, 1.8
	Acne	7489	11.3	10.8, 11.8	-0.5	-0.1	-1.5, 1.3	0.1	0.6	-1.7, 3.0	-0.5	-0.2	-3.3, 2.9
	Menarche	7481	12.7	12.4, 13.1	-1.1	-0.6	-1.5, 0.3	-0.7	-0.2	-1.8, 1.3	-0.7	-0.4	-2.2, 1.5
	Combined estimate	7481				-1.1	-1.9, -0.2		-0.9	-2.2, 0.4		-0.9	-2.8, 1.0

ICS: inhaled corticosteroids. INCS1: first-generation intranasal corticosteroids. Diseased refers to asthma, allergy, skin diseases or muscle and joint diseases. Treatment refers to treatment for the disease without glucocorticoids.
